# “Freedom and Dignity Are Worth More than Life”: The Dramatic Suicide of an Anti-Vax Man

**DOI:** 10.3390/healthcare10112141

**Published:** 2022-10-28

**Authors:** Sara Sablone, Lorenzo Spagnolo, Enrica Macorano, Mauro Claudio Ciavarella, Natascha Pascale, Giuseppe Strisciullo, Francesco Introna, Aldo Di Fazio

**Affiliations:** 1 Section of Legal Medicine, Interdisciplinary Department of Medicine, University of Bari, 70124 Bari, Italy; 2 Public Health Unit, Local Health Authority of Brindisi, 72100 Brindisi, Italy; 3 Regional Complex Intercompany Institute of Legal Medicine, 85100 Potenza, Italy

**Keywords:** anti-vax, suicide, forensic pathology, forensic psychiatry

## Abstract

Since the beginning of the COVID-19 public health emergency, we have witnessed an increase in psychiatric problems and pathologies, such as depression, anxiety, isolation, posttraumatic stress disorder, substance abuse, and burnout. The world’s collective sentiment finally turned toward optimism after authorization was granted for the COVID-19 vaccines’ emergency use by the FDA in December 2020. With the increase in vaccine coverage in Western countries, case counts and deaths gradually plummeted while activity restrictions were progressively lifted. At the same time, however, a new COVID-19-related public health issue has arisen, as a substantial number of eligible individuals refused vaccination. Behaviors assumed by the so-called anti-vax people in manifesting their own opposition towards COVID-19 vaccination are various, and sometimes assume the forms of dramatic gestures with symbolic value, such as suicide. Here, we present the case of a healthy, convinced anti-vax, 58-year-old man, who allowed himself to be run over by a moving train in the presence of eyewitnesses, bringing with him a demonstrative note of his reasons. The present article aims to raise awareness against the social and psychological impact of COVID-19 vaccination refusal and to point out the need of a specific support net to avoid the spread of psychological impairment, social isolation and suicidal behaviors among the “anti-vax community”.

## 1. Introduction

The COVID-19 pandemic has resulted in unprecedented social and economic disruptions, with a profound worldwide impact on public health and lifestyle [1,2]. Given the limitations of existing treatment for COVID-19, since the beginning of the pandemic all hopes for its effective control have been focused on the development of a vaccine against SARS-CoV-2 [3]. Thus, the COVID-19 vaccine is a crucial matter of public health, as it is considered our main hope in facing the virus (and its variants) and to return to everyday life [4].

Indeed, there is no doubt about the effectiveness of the vaccine, in addition to other infection control measures, such as surface/environment disinfection practices, hand hygiene, physical and social distancing, and the use of personal protective equipment [5,6,7,8,9,10,11]. However, herd immunity—estimated to occur when a large part of the population is covered—is highly dependent on individuals’ willingness to be vaccinated, although in the specific case of COVID-19, the concept of classical herd immunity may not apply [12,13]. Therefore, vaccine hesitancy was one of the major concerns among health authorities, even before the vaccine’s approval by the Food and Drug Administration (FDA) or the European Medicines Agency (EMA) [14].

According to MacDonald et al., “vaccine hesitancy” can be defined as the “delay in acceptance or refusal of vaccination despite availability of vaccination services. Vaccine hesitancy is complex and context specific, varying across time, place and vaccines. It is influenced by factors such as complacency, convenience and confidence.” Thus, vaccine hesitancy cannot be generalized and should be considered a multidimensional phenomenon occurring in a specific environment in which crucial determinants are factors such as complacency, convenience, and confidence. Moreover, even contextualizing to one’s environment, such factors vary over time and according to the vaccine itself, complicating the interpretation of the whole picture. Complacency means the low perception of disease risk; hence, vaccination seems unnecessary. Confidence denotes trust in the vaccination safety, effectiveness, and competence of healthcare systems. Convenience involves the availability, affordability, and delivery of vaccines in a comfortable context. Several determinants modify vaccine hesitancy and determine whether to refuse, delay, or accept some or all vaccines. These include contextual influences which arise from historical, socioeconomic, cultural, ecological, health system, and political factors [15].

Numerous COVID-19 vaccination studies have documented an association between these factors and the acceptance or refusal of the COVID-19 vaccine [16,17,18].

Moreover, the national lockdown context may also have influenced people’s intention of being vaccinated because of misinformation and the emerging concerns of governments across Europe referring to beliefs about rushed vaccine development [19,20]. 

The result of this complex situation, and of the “infodemic” that derives from the many subjects and interests involved, determined the radicalization of certain convictions—a shift towards positions or solutions beyond any compromise or mediation—that resulted in the formation of “anti-vax communities”. 

Burgess et al. make a clear distinction between vaccine hesitancy and anti-vax sentiments and community [21,22]. Indeed the latter *“proactively opposes vaccinations denying the existence of COVID-19 or ascribing bizarre, deliberately malignant biopsychosocial effects to current vaccines and boosting trust in fake and irrational beliefs*” [23].

In such meaning, other studies warn against the risk of considering the anti-vax movement as a cult. Indeed, negative labels towards cults and sects may promote further separation from the rest of the community, creating martyrs and strengthening new beliefs, thus encouraging further involvement in the movement [24].

However, it cannot be denied that the anti-vax movements represent a danger for society as a whole. They both promote vaccine hesitancy and fuel the expression of radical positions through extreme gestures such as demonstrative suicides, violent street protests, intimidation and violence against health workers, public administration, and scientists, as often reported in press and found during our forensic activity.

Here, we present the case of a healthy, convinced anti-vax, 58-year-old man, who allowed himself to be run over by a moving train in the presence of eyewitnesses, bringing with him a demonstrative note of his reasons.

## 2. Case Report

### 2.1. The Accident Site Inspection 

On a cold January afternoon in 2022, the railway police alerted our Institute of Legal Medicine of the occurrence of a railway investment near a train station.

The authors of the present paper had been commissioned by the local law enforcement for crime scene investigation. Upon our arrival at the site, investigators told us that a man reached the railway by his bike and remained on the spot, declaring his will to kill himself. Despite people in the station shouting at him to move away, the man was eventually hit by the upcoming train travelling at a speed of about 150 km/h.

**Figure 1:**
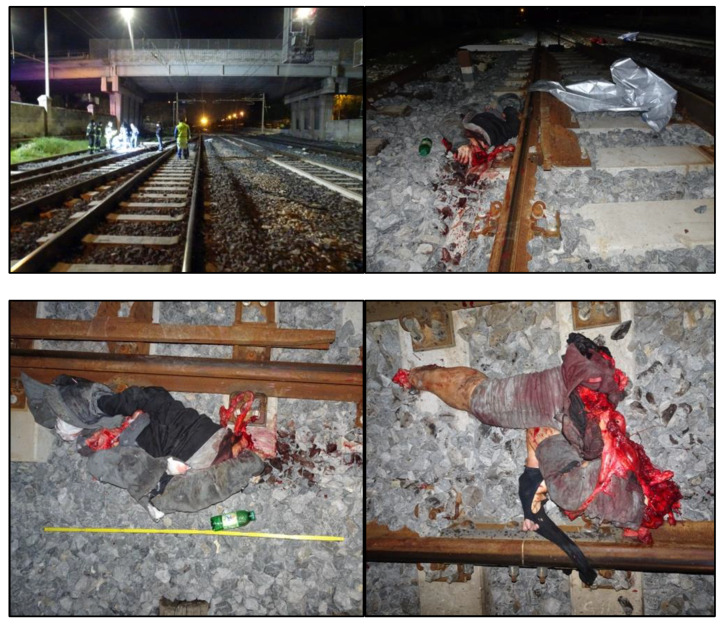
The railroad track where the investment occurred, with the man’s body segments lying near the impact point.

The man’s body laid across the railroad track and was extensively segmented, with the projection of blood, bone fragments, and soft tissue pieces along several hundred meters of the track itself (Figure 1). 

The judicial authority asked for the corpse forensic examination, the pieces of which were then collected and moved from the investment site to the Institute of Legal Medicine in Bari. 

### 2.2. The External Examination of the Corpse

**Figure 2:**
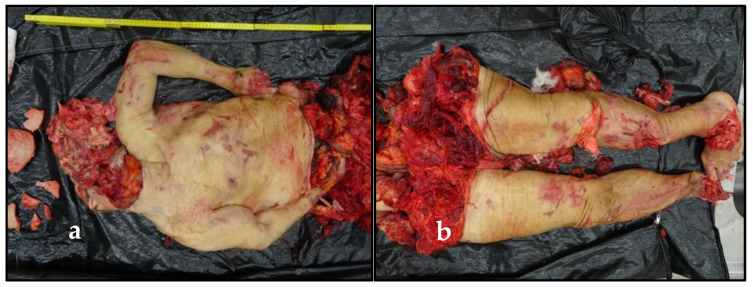

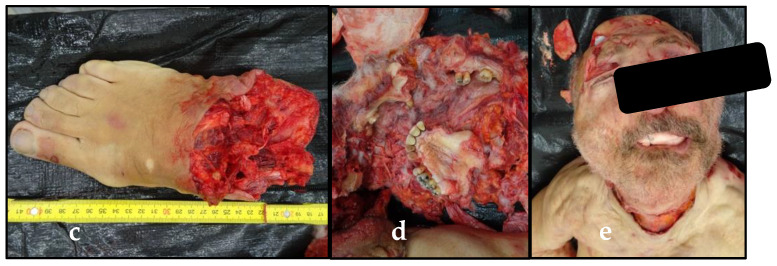
The widespread depletion of the corpse: (**a**) the neck, the trunk, and the arms; (**b**) the pelvis and the lower limbs; (**c**) the right foot; (**d**) some neurocranium and face’s soft tissues pieces, and some dental elements; (**e**) the victim’s face, whose soft tissues were reassembled and supported by packing a puppet.

Preliminarily, a careful external examination was performed. Rigor and algor mortis were affected by the widespread corpse depletion. Hypostases were not appreciable because of the complete body exsanguination. The corpse showed multiple, large, and deep injuries. The edges of the wounds were well defined and bruised, and underlying muscles and organs were widely exposed or also sharply cut. Overall, two body segments were recognizable: the first segment included some neurocranium and soft tissues pieces of the face, some dental elements, the neck region, the trunk, and the arms. The second one included the pelvis and the lower limbs. Then, there was a detached right foot (Figure 2). 

Despite the skull-brain bursting, the eyeballs were present and intact. Thus, vitreous humor samples and a fragment of liver parenchyma have been collected to perform toxicological investigations. 

**Figure 3:**
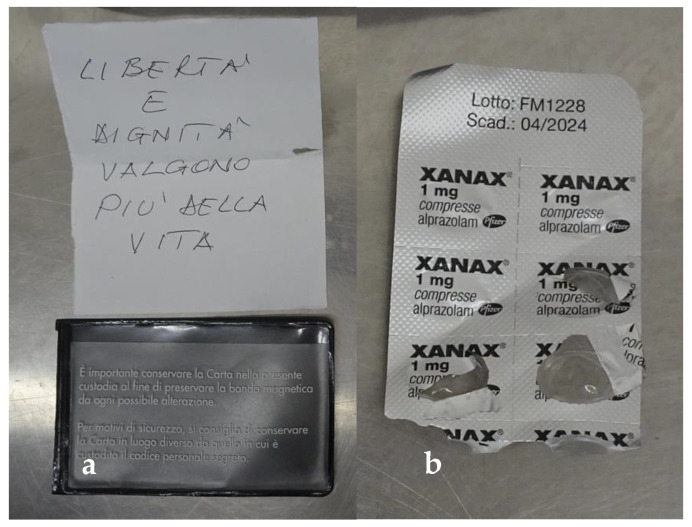
Personal items found in the victim’s clothing pockets: (**a**) the suicide note (**b**) the blister with three pills of a tranquilizer (Xanax, Alprazolam).

Then, the victim’s clothing was carefully inspected. A blister with three pills of a tranquilizer (Xanax, Alprazolam) was found as well as a plastic document holder containing a small sheet of paper with the following handwritten note: “freedom and dignity are worth more than life” (“libertà e dignità valgono più della vita”) (Figure 3). 

### 2.3. Toxicological Findings

Screening toxicological tests on a vitreous humor sample taken from the dead man’s eyeballs have been carried out to verify the presence of exogenous substances such as drugs or alcohol. The headspace gas chromatographic method for ethyl alcohol detection and the immunochemical method for methadone, cannabinoids, cocaine, opiates, barbiturates, benzodiazepines, amphetamines, and tricyclic antidepressants detection gave negative results. The subsequent qualitative investigation on the cadaveric blood extracted from the liver tissue samples confirmed the absence of the above-mentioned substances. Therefore, the man was not in a state of acute intoxication by exogenous substances at the time of the railway investment.

Ultimately, all circumstantial, necroscopic, and laboratory data converged on the diagnosis of suicidal death from traumatic shock secondary to railway investment.

### 2.4. The Corpse Identification

Although some cadaveric tissues had been cautiously sampled for identification purposes, the railway police were informed about a missing person complaint from two men, who had reported their brother’s disappearance 24 h previously.

The complainants reached the Institute of Legal Medicine in Bari to view the victim’s clothing and personal objects, which were recognized as belonging to their missing brother. Then, they recognized the victim’s face, whose soft tissues were preliminarily reassembled. 

The brothers reported that the man lived alone, that he was psycho-physically healthy, completely autonomous, and with a fairly active social life. They added that their brother had been deeply shocked by the restrictions imposed on people not voluntarily undergoing anti-COVID-19 vaccination, and that he had refused to get vaccinated in order not to succumb to a “health dictatorship”. His choice no longer allowed him to freely live daily life, forcing him into social isolation. Relatives claimed that the victim must have only recently started taking tranquilizers, as they often saw their brother and could rule out that he was suffering from evident mental illness or had previously taken psychotropic drugs.

## 3. Discussion

The COVID-19 pandemic determined relevant effects on mental health in relation to the disruptions it directly or indirectly caused in the socio-economic tissue of the global community [25,26]. These aspects have been clearly demonstrated by a number of studies which pointed out the negative influence of the pandemic on the psychological well-being of people around the world, especially in the more fragile parts of society [27,28]. 

Indeed, the negative effects on mental health are not equally shared among all sections of the population. The elderly, especially those institutionalized, unemployed people, children and adolescents are undoubtedly the groups most at risk. The same could be said for essential workers, especially health care providers, due to a greater risk of contagion, work intensification and staff shortages [29,30,31,32,33]. More vulnerable groups also include immigrants, refugees, indigenous communities, and low-wage workers [34].

Moreover, it has been noted that survivors of COVID-19 are most at risk of developing mental health problems due to the possible neurotropism, immune response to SARS-CoV-2, neuroinflammation, hyperactivity of the adrenal axis, disruptions of neuronal circuits, and neuronal loss [35,36,37].

Therefore, especially in the first phase of the pandemic, there was a significant increase in all psychiatric pathologies and suicide also involving subjects not directly affected personally or in the family by the disease [38,39].

However, in the current phase, the remaining—but not small—population still stubbornly rejecting the vaccine risk experiencing new heavy restrictions, both in the occupational setting and in the social field, depending on the course of the pandemic during winter.

This would lead, as happened for the general population in the most difficult phases of the pandemic, to an increased risk of social isolation, radicalization of extremist groups and fallacious beliefs fueled by misleading information largely expressed on social networks or through private groups of the anti-vax community on the internet [40,41].

The discrimination and stigma associated with vaccination refusal, together with fear of illness and uncertainty about the future would precipitate anxiety- and stress-related disorders, causing relevant social and psychological distress on those still refusing COVID-19 vaccination, as is already happening, with a possible rise in antisocial behaviors. Indeed, as happened in the present case, refusal of vaccination has caused an otherwise healthy person to become substantially isolated from society, ultimately leading to self-induced research apparently confirming false and misleading information that eventually caused an anxious state and death from suicide.

Anti-vax people could therefore become the population group most at risk for the onset of psychiatric disorders, especially at the present time when the pandemic seems to be coming to an end.

Thus, the need for a specific support net to avoid the spread of psychological impairment, social isolation, and suicidal behaviors among the “anti-vax community”.

Selective screening, prophylactic psychiatry, and psychoeducational interventions have proven to be evidence-based and cost-effective preventive interventions which could help to avoid gestures such as the one in the presented case.

Moreover, a strategy to decrease inequalities and to remodulate communication pattern is needed in order to psychologically protect fragile subjects and ultimately improve vaccination plan.

## 4. Limitations of the Study

In order to fully understand the context in which the insane act took place, we repeatedly tried to contact the relatives of the deceased. However, after an initial acceptance of the meeting, the family refused to be heard because they *“did not feel able to relive that moment of great suffering”*. Therefore, we were not able to fully reconstruct the family pattern of the victim.

## Data Availability

Not applicable.
